# Antidiabetic effects of a lipophilic extract obtained from flowers of *Wisteria sinensis* by activating Akt/GLUT4 and Akt/GSK3β

**DOI:** 10.29219/fnr.v64.3589

**Published:** 2020-09-07

**Authors:** Yibing Lv, Wenjie Ren, Yirui Zhang, Yun Huang, Ji Hao, Kun Ma, Yuanren Ma, Xinzhou Yang

**Affiliations:** School of Pharmaceutical Sciences, South-Central University for Nationalities, Wuhan, China

**Keywords:** antidiabetes, GLUT4, dyslipidemia, hyperglycemia, insulin resistance

## Abstract

**Background:**

Type 2 diabetes mellitus is primarily caused by insulin resistance (IR) in insulin-sensitive tissues, including liver, white adipose tissues (WAT), and skeletal muscles. Discovering nutritious foods with antidiabetic effects is of great significance. Numerous published reports indicated that protein kinase B (Akt) and glucose transporter 4 (GLUT4) play crucial roles in ameliorating IR and diabetic symptoms.

**Objective:**

In the present study, antidiabetic effects and the potential mechanism of action of WS-PE (a lipophilic extract from edible flowers of *Wisteria sinensis*) were explored with L6 cells *in vitro* and in high-fat diet (HFD) + Streptozocin (STZ)-induced diabetic mice *in vivo*.

**Design:**

*In vivo*, HFD + STZ-induced diabetic mice were used as diabetic models to investigate the potential antidiabetic and antidyslipidemic activities. *In vitro*, a novel GLUT4 translocation assay system was established to evaluate the potential effects of WS-PE on GLUT4 translocation. Western blot analysis was adopted to investigate the molecular mechanisms of WS-PE both *in vivo* and *in vitro*.

**Results:**

*vitro*, WS-PE increased glucose uptake by stimulating GLUT4 expression and translocation, which were regulated by Akt phosphorylation. *In vivo*, the WS-PE treatment ameliorated the hyperglycemia, IR, and dyslipidemia and reversed hepatic steatosis and pancreatic damage in diabetic mice. The WS-PE treatment increased GLUT4 expression by Akt activation in WAT and skeletal muscle. Akt activation stimulated GSK3β phosphorylation in liver and skeletal muscles, indicating that WS-PE showed regulatory effects on glycogen synthesis in liver and skeletal muscles.

**Conclusion:**

These *in vitro* and *in vivo* results indicated that the WS-PE treatment exerted antidiabetic effects by activating Akt/GLUT4 and Akt/GSK3β.

## Popular scientific summary

- Flowers of *Wisteria sinensis* form a popular food material for good health in China. It is important to explore the antidiabetic effects and the underlying mechanisms of the effects of this food, which will contribute to promoting the consumption of this food and protecting human health.
- The results of this study indicated that WS-PE exerted antidiabetic effects by activating Akt/GLUT4 and Akt/GSK3β and further ameliorating insulin resistance, hyperglycemia, and dyslipidemia.
- Flowers of *W. sinensis* having antidiabetic effects deserve large-scale production and utilization.

Diabetes mellitus (DM), a chronic metabolic disease that affects individuals worldwide, is characterized by glucose and lipid metabolism disorders and insulin secretion deficiency (1, 2). According to the statistics estimated by the International Diabetes Federation (IDF), about 451 million adults suffered from diabetes around the world in 2017, and 693 million adults are predicted to be affected by diabetes by 2045 (3). DM can be divided into type 1 diabetes mellitus (T1DM), which is characterized by absolute insulin secretion dysfunction, and type 2 diabetes mellitus (T2DM), which is characterized by insulin resistance (IR) and relatively insufficient insulin secretion. Statistically, more than 90% diagnosed DM patients have T2DM (4). T2DM is a chronic disease that presents with typical symptoms of hyperglycemia and IR (5). Presently, doctors usually use insulin injection combined with several oral antidiabetic agents to treat T2DM; however, no ideal therapeutic effects have been noted because of the lack of safety, the limitations of adverse effects, and drug dependence (6). Thus, there is a strong demand for the development of more effective and safer antidiabetic agents with limited side effects. Natural functional foods, which are characterized by safety, satisfactory effectiveness, and few side-effects, have attracted increasing interest (7, 8). Previous reports have shown that several functional foods, such as bitter gourd, avocado pear, and broccoli, have remarkable antidiabetic effects (9–11). These foods may influence the currently available therapies and enhance their effects.

*Wisteria sinensis* (Sims) Sweet, a climbing plant belonging to the Leguminosae family, is available all over China (12). The flowers of *W. sinensis* are used in many traditional Chinese cuisines, including the Chinese flower cake and Chinese flower jam. The extracts from plants belonging to the *genus Wisteria* reportedly have anti-oxidant, anti-tumor, and anti-inflammatory activities (13–15). In our knowledge, this is the first investigation to elucidate the potential antidiabetic activity of *W. sinensis*.

Glucose transporter 4 (GLUT4) is one of the most appealing targets for treating T2DM, and the translocation of GLUT4 and the expression levels of GLUT4 are related to systemic insulin-mediated glucose homeostasis (16). Currently, the GLUT4 translocation assay system based on L6 cells with stable over-expression of insulin-responsive aminopeptidase (IRAP)-mOrange has been established. This system has been used for screening potential antidiabetic agents among foods (7, 8, 17, 18). Based on the bioassay method, a lipophilic extract from the flowers of *W. sinensis* (WS-PE) was found to positively stimulate GLUT4 translocation and increase glucose uptake. The preliminary result suggests a hypothesis that WS-PE may have potential antidiabetic effects. The assumption was *proven* with L6 cells *in vitro* and in diabetic mice *in vivo*. Moreover, proteins that regulate lipid and glucose metabolism were examined to reveal the molecular mechanisms of WS-PE onT2DM.

## Materials and methods

### Reagents

Fetal bovine serum (FBS, SH30396.03), antibiotics (100 U/mL penicillin and 100 μg/mL streptomycin, J170042), and α-minimum essential medium (α-MEM, SH30265.01B) were purchased from Hyclone (Logan, UT, USA). The insulin enzyme-linked immunosorbent assay (ELISA) kit (H203) was obtained from the Jiancheng Bioengineering Institute (Nanjing, Jiangsu Province, China). Streptozocin (STZ) was purchased from Sigma-Aldrich Company (S0130, St. Louis, Missouri, USA). Triglycerides (F001, TG), total cholesterol (F002, TC), free fatty acid (A042-2, FFA), high-density lipoprotein cholesterol (A112-2, HDL-C), and low density lipoprotein cholesterol (A113-1, LDL-C) kits were purchased from Jiancheng Bioengineering Institute. The 2-[N-(7-nitrobenz-2-oxa-1,3-diaxol-4-yl)amino]-2- deoxyglucose (2-NBDG) assay kit was purchased from Cayman Chemical (11046, Ann Arbor, Michigan, USA). The BCA (bicinchoninic acid) protein quantification kit was purchased from Beyotime Biotechnology (P0012, Nantong, Jiangsu Province, China). Antibodies of β-actin (3700S), GLUT4 (2213), p-GSK3β (ser9, 9323T), GSK3β (9832S), Akt (4691S), p-Akt (ser473, 4060), and the corresponding secondary antibodies (Rabbit Anti-Mouse Ig G mAb, 58802S; Mouse Anti-Rabbit IgG mAb, 93702S) were obtained from Cell Signaling Technology (Danvers, MA, USA). Enhanced chemiluminescence kits (BWR1064) were obtained from Chongqing Biospes Co., Ltd.

### Plant material and preparation of WS-PE

In April 2017, lab associates collected the flowers of *W. sinensis* in the campus of South-Central University for Nationalities (SCUN), Wuhan, China. Professor Dingrong Wan, who worked in the School of Pharmaceutical Sciences, SCUN, identified the specimens as flowers of *W. sinensis* (Sims) Sweet. The voucher specimen (SC0095) has been deposited in the School of Pharmaceutical Sciences, SCUN, Wuhan, China. Herein, mashed air-dried flowers of *W. sinensis* (500 g) were immersed in 80% ethanol (4× 3 L, 3 d each) to obtain extracts at room temperature. The solvents were evaporated under reduced pressure, which yielded 50 g of residue. The residue was mixed with water in a ratio of 1:20, and then, petroleum ether (4 × 1.0 L), ethyl acetate (4 × 1.0 L), and *n*-BuOH (4 × 1.0 L) were added in that order to extract the slurry. The solvents were evaporated under reduced pressure, which yielded 6 g petroleum ether extract (WS-PE), 7 g ethyl acetate extract, and 22 g *n*-BuOH extract, all of which were subsequently used to evaluate GLUT4 translocation.

### Cell culture, glucose uptake test, and GLUT4 translocation assay

Detailed methods of cell culture and glucose uptake test were consistent with our previous reports (7, 8). Insulin-stimulated GLUT4 and IRAP had a strong colocalization in cellular GLUT storage vesicles (GSVs). The translocation of GLUT4 could be assessed by detecting the IRAP (19). Methodology validation is shown in the Supplementary File. α-MEM supplemented with 10% FBS and 1% antibiotics (100 U/mL penicillin and 100 μg/mL streptomycin) was used to culture L6 cells stably expressing IRAP-mOrange (L6 IRAP-mOrange) at 37 °C in 5% CO_2_. At the beginning of the experiment, L6 IRAP-mOrange was cultured in a 48-well plate and incubated until 100% confluence was reached, followed by 2 h starvation with serum-free α-MEM. A dose of 30 μg/mL of WS-PE was used to treat L6 cells, and insulin (100 nM) was used as the positive control. The same solvent of WS-PE was used to treat L6 cells as a normal control. A laser scanning confocal microscope, LSM 510 (Carl Zeiss, Jena, Germany), was used to image cells and monitor the dynamic translocation of IRAP-mOrange. The photos were taken after the addition of samples by using an excitation laser wavelength of 555 nm every 5 min for 30 min.

### Animals and treatments

Eight-week old male C57BL/6j mice (*n* = 60) were purchased from Beijing HFK Biosciences Co., Ltd. and maintained under a 12 h light/dark cycle in a controlled environment at 23 ± 2°C with a humidity level of 55 ± 10%. All mice had access to food and water *ad libitum*. After 1 week of adaptive feeding, mice were initially divided into two groups. Mice in the normal control group (NC, *n* = 8) were fed with a normal chow diet (1022, Beijing HFK Bioscience Co., Ltd), whereas other mice (*n* = 52) with T2DM which comprised the intervention group were fed with a high-fat diet (HFD) (H10045, Beijing HFK Bioscience Co., Ltd., China). The HFD contained 4.73 kcal per gram of food, of which 20% kcal were obtained from proteins, 35% kcal from carbohydrates, and 45% kcal from fats. After 4 weeks of HFD administration, mice were induced to develop obesity, dyslipidemia, and IR. Then, the fat mice were intraperitoneally injected with 40 mg/kg streptozotocin citrate buffer solution (pH 4.5), and also received injections twice for 2 consecutive days. After the second injection, fasting blood glucose (FBG) levels of mice were measured every 3 days over 12 days. All blood glucose data were recorded seriously. During four FBG tests, mice that maintained high FBG levels (FBG level **§[** 11.1 mmol/L) were considered successful diabetic models. The mice that proved to be successful for modeling diabetic mice were randomly divided into four groups (each group containing eight mice): the diabetic control group (DC), low-dose WS-PE treatment group (WPL, 80 mg/kg/day), high-dose WS-PE treatment group (WPH, 160 mg/kg/day), and metformin-treated group (MET, 200 mg/kg/day). WS-PE was dissolved in saline containing 0.5% CMC-Na. NC and DC groups were orally administered the saline containing 0.5% CMC-Na. All groups were orally administrated their respective treatments every day for 4 weeks, and all mice in different groups received gavage at a dose of 0.1 mL of liquor per 10 g of body weight of mice. FBG levels and body weights of mice were tested weekly. On the 26th day of the treatment, the oral glucose tolerance test (OGTT), which is an indicator of IR and glucose homeostasis, was conducted in 12 h fasted mice from all groups. Mice in all groups were orally administered glucose[AQ1] at a dose of 2 g/kg. After glucose administration, 0, 30, 60, 90, and 120 min were selected as the key time points to monitor blood glucose levels using a blood glucose meter (One Touch Ultra, Lifescan Inc., Wayne, USA). The data based on OGTT results were used to generate areas under the curve (AUC), which reflected ameliorated damage glucose tolerance directly.

### Histology, immunohistochemistry and biochemical analysis of serum

The methods of histology and immunohistochemistry were in line with our previous reports (8). At the end of experiment, mice from all groups received retro-orbtial sinus puncture under diethyl ether anesthesia to collect blood samples. The blood samples were centrifuged at 3,000 g for 15 min to separate and collect the serum. An automatic biochemical analyzer (Hitachi 7180+ISE, Tokyo, Japan) was used to test all serum biochemical parameters including total cholesterol (TC), triglycerides (TG), aspartate aminotransferase (AST), alanine aminotransferase (ALT), low-density lipoprotein cholesterol (LDL-C), and high-density lipoprotein cholesterol (HDL-C). A serum-free fatty acid (FFA) assay kit was used to detect FFA. A rodent insulin ELISA kit was used to measure serum insulin content.

### Protein extracts and Western blotting

Proteins in L6 cells, skeletal muscles, liver, and white adipose tissues (WAT) were extracted according to previously reported methods (20). Briefly, L6 cells (8 × 10^5^ cells) were cultured into 100 mm dishes for 7 days to induce myotubes in 3 mL of α-MEM with 2% FBS. Then, insulin (100 nM), WS-PE (10, 20 and 30 μg/mL), or normal control (0.1% DMSO Dimethyl sulfoxide ) were used to treat the L6 myotubes for 12 h. The cells were then washed with cold PBS. Next, ice-cold RIPA buffer [50 mM Tris-HCl (pH 7.4), 150 mM NaCl, 1% NP-40, 0.1% SDS] containing protease inhibitor cocktail (Roche, Basel, Switzerland) and phosphatase inhibitor cocktail (Selleckchem, Houston, USA) was used to crack cells. The whole-cell lysate was centrifuged at 15,000 *g* for 10 min to remove insoluble materials. A BCA (bicinchoninic acid) protein assay kit was used to determine the protein concentration.

Frozen skeletal muscle, liver, and WAT were returned to room temperature, weighed, grinded, and mixed with ice-cold RIPA buffer containing protease inhibitor cocktail and phosphatase inhibitor cocktail. The mixture was lysed for 30 min on ice and centrifuged at 15,000 *g* for 15 min at 4°C. Then, all insoluble material was discarded and the BCA kit was used to measure the protein concentration. The proteins collected from tissues and cells were subjected to Western blot analysis according to the previously described methods (21).

### Statistical analysis

Differences between groups were analyzed by one-way analysis of variance (ANOVA). Data were shown as means ± standard error of mean. Tukey’s post hoc test of GraphPad Prism 5.0 software packages was used to perform statistical analyses. A value of *P* < 0.05 was considered as statistically significant.

## Results

### WS-PE stimulated GLUT4 expression and translocation to enhanced glucose uptake in L6 cells

**Fig. 1:**
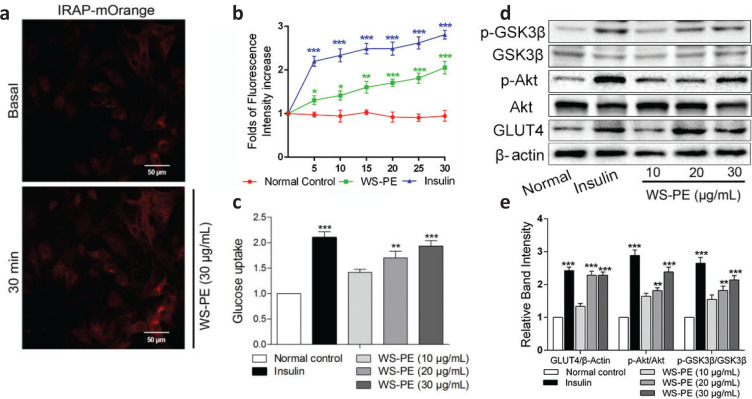
Preliminary screening of WS-PE *in vitro*. (a) WS-PE (30 μg/mL) increased IRAP fluorescence intensity within 30 min. Scale bar, 50 μm. (b) WS-PE induced fluorescence intensity increasing within 30 min, and insulin increased fluorescence intensity. as a positive control. ^*^ *P* < 0.05, ^**^ *P* < 0.01, ^***^ *P* < 0.001 compared with normal control. (c) Different doses of WS-PE enhanced glucose uptake in a dose-dependent manner. ^**^ *P* < 0.01, ^***^ *P* < 0.001 compared with normal control. (d) WS-PE stimulated GLUT4 expression and phosphorylation of Akt and GSK3β in L6 myotubes. (e) Relative band intensity of GLUT4, p-Akt and p-GSK3β. Data are shown as means ± SEM, shown as relative band intensity compared with normal control. ^**^ *P* < 0.01, ^***^ *P* < 0.001 compared with normal control. WS-PE: a lipophilic extract from flowers of *Wisteria sinensis*.

Fig. 1a, b show that 30 μg/mL of WS-PE increased the fluorescence intensity to 1.92 folds compared with normal control in a time-dependent manner, indicating that the WS-PE treatment strongly stimulated GLUT4 translocation in L6 myotubes. Furthermore, WS-PE also enhanced glucose uptake in a dose-dependent manner, and 30 μg/mL WS-PE increased the glucose uptake to 2.0-fold compared with the normal control (Fig. 1c). Moreover, the results of Western blot analysis showed that WS-PE increased GLUT4 expression and stimulated Akt and GSK3β phosphorylation in L6 myotubes (Fig. 1d, e).

### WS-PE treatment ameliorated hyperglycemia and body weight loss and improved glucose tolerance in diabetic mice

**Fig. 2:**
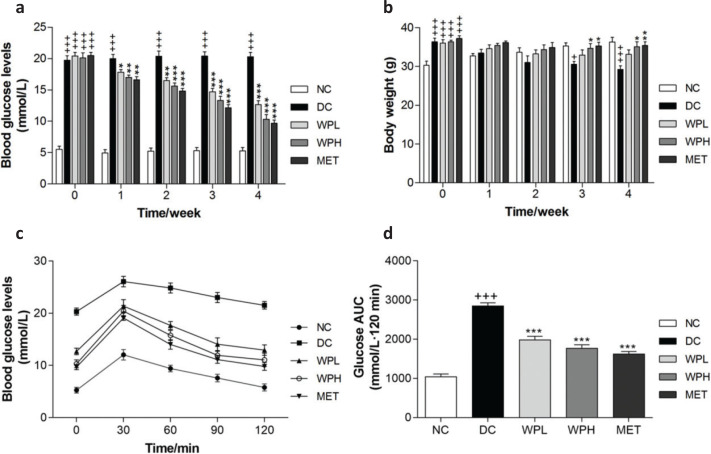
The WS-PE treatment ameliorated hyperglycemia, glucose tolerance, and body weight loss in diabetic mice. (a) WS-PE and metformin ameliorated high FBG levels. (b) WS-PE regulated body weight loss of diabetic mice during 4 weeks treatment. (c) Effects of WS-PE on reversing damaged glucose tolerance. (d) AUC analysis of diabetic mice OGTT. Data are means ± SEM (*n* = 8). ^+++^ *P* < 0.001 compared with NC group, ^*^ *P* < 0.05, ^**^ *P* < 0.01, ^***^ *P* < 0.001 compared with DC group. NC: normal control group. DC: diabetic control group. WPL: a low-dose of WS-PE-treated group. WPH: a high-dose of WS-PE-treated group. MET: metformin-treated group.

Diabetic mice showed decreased body weight after STZ injection (Fig. 2b). During the 4 weeks of continuous oral administration of WS-PE, body weight loss of diabetic mice in the WS-PE-treated group was significantly ameliorated; however, body weight of diabetic mice in the DC group decreased sharply. Before the first week of treatment, diabetic mice showed higher blood glucose levels than mice in NC group mice. The WS-PE or metformin treatment significantly ameliorated hyperglycemia in diabetic mice when compared with DC group mice (Fig. 2a).

On the 26th day of the experiment, an OGTT experiment was conducted to verify the effects of WS-PE on improving the damaged glucose tolerance. Diabetic mice in the DC group had damaged glucose tolerance, supported by the fact that their blood glucose levels increased sharply and were maintained at higher levels even at 120 min. However, mice treated with MET or WS-PE showed that FBG levels declined gradually, indicating that WS-PE reversed the damaged glucose tolerance (Fig. 2c, d).

### WS-PE treatment ameliorated serum insulin levels and dyslipidemia in diabetic mice

**Fig. 3:**
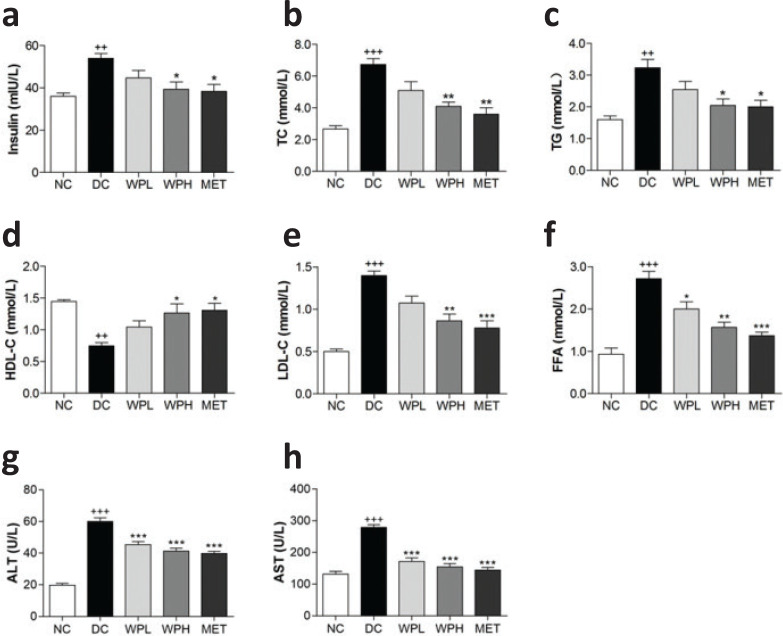
The WS-PE treatment improved abnormal serum insulin and dyslipidemia. (a) WS-PE decreased the serum insulin level in diabetic mice. (b–f) WS-PE decreased serum TC, TG, LDL-C, FFA, ALT, and AST levels and increased HDL-C level. Data are means ± SEM (*n* = 8). ^++^ *P* < 0.01, ^+++^ *P* < 0.001 compared with the NC group, ^*^ *P* < 0.05, ^**^ *P* < 0.01, ^***^ *P* < 0.001 compared with the DC group. NC: normal control group. DC: diabetic control group. WPL: a low-dose of WS-PE-treated group. WPH: a high-dose of WS-PE-treated group. MET: metformin-treated group.

The serum insulin levels and serum lipid indices in all mice were tested at the end of the experiments. Serum insulin levels of diabetic mice were much higher in the DC group than in the NC group. Four-week continuous treatment with WS-PE or metformin gradually decreased the high serum insulin levels of diabetic mice (Fig. 3a).

Moreover, serum lipid indices of diabetic mice in DC group, including serum TG, TC, ALT, AST, FFA, LDL-C, and HDL-C, showed abnormal levels, which indicated that diabetic mice had symptoms of dyslipidemia. However, the WS-PE treatment decreased high levels of TC, TG, FFA, AST, ALT and LDL-C, and increased the level of HDL-C in diabetic mice (Fig. 3b–h), suggesting that the WS-PE treatment improved the abnormal lipid metabolism of diabetic mice. Moreover, the WS-PE treatment decreased high levels of ALT and AST, and this indicated that WS-PE ameliorated damage due to liver steatosis.

### Histological analysis of the liver and pancreas

**Fig. 4:**
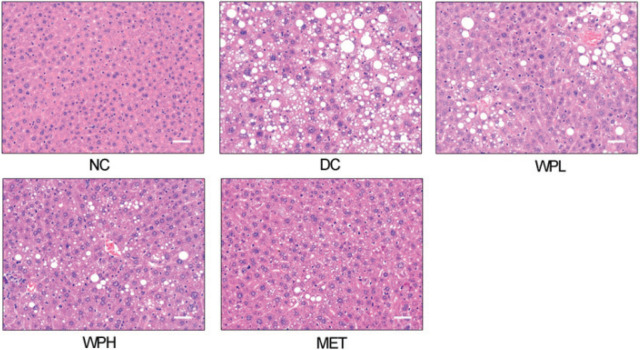
Hepatic steatosis in diabetic mice was resolved by the WS-PE treatment. HE staining of livers from mice. Scale bar, 50 μm. NC: normal control group. DC: diabetic control group. WPL: a low-dose of WS-PE-treated group. WPH: a high-dose of WS-PE-treated group. MET: metformin-treated group.

Histological examination of the liver showed that diabetic mice in the DC group suffered from sickly steatosis containing empty vacuoles. Mice in the WS-PE- or metformin-treated group showed ameliorated hepatic steatosis with decreased adipose vacuoles, indicating that 4-week treatment with WS-PE or metformin reversed the severe hepatic steatosis (Fig. 4).

**Fig. 5:**
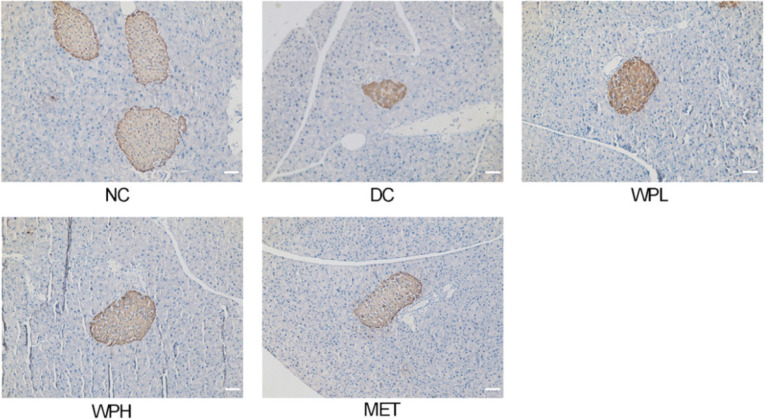
The WS-PE treatment reversed the damaged pancreas. Scale bar, 50 μm. NC: normal control group. DC: diabetic control group. WPL: a low-dose of WS-PE-treated group. WPH: a high-dose of WS-PE-treated group. Met: metformin-treated group.

Immunohistochemical observations of the pancreas in the NC group mice showed normal islet morphology and strong insulin-immunoreactivity of β-cells in pancreatic islets. However, diabetic mice in the DC group showed reduced area of insulin-immunoreactive β-cells. WS-PE- or metformin-treated mice showed a significantly greater area of insulin-immunoreactive β-cells than DC group mice (Fig. 5). These results indicated that the WS-PE treatment protected impaired pancreatic β-cells and damaged pancreatic islets in diabetic mice.

### WS-PE stimulated expression of GLUT4 in WAT and skeletal muscles

**Fig. 6:**
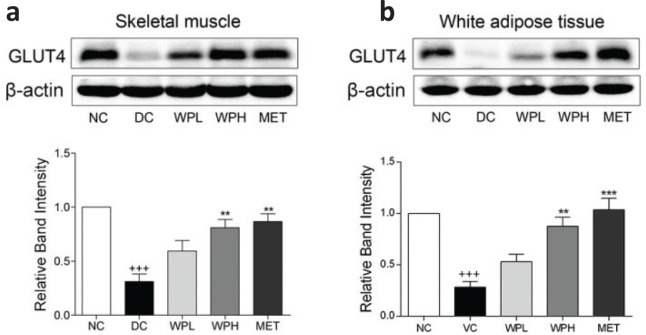
The WS-PE treatment stimulated GLUT4 expression in skeletal muscles and WAT. Western blot analysis and relative band intensity of GLUT4 in skeletal muscles (a) and WAT (b). Data are means ± SEM, shown as relative band intensity compared with NC group (*n* = 3). ^+++^ *P* < 0.01 compared with NC group, ^**^ *P* < 0.01 compared with DC group. NC: normal control group. DC: diabetic control group. WPL: a low-dose of WS-PE-treated group. WPH: a high-dose of WS-PE-treated group. MET: metformin-treated group.

GLUT4 expression levels in skeletal muscles and WAT in the DC group were lower than those in the NC group (Fig. 6). Interestingly, 4-week treatment with WS-PE or metformin increased the expression of GLUT4 in skeletal muscles (Fig. 6a) and WAT (Fig. 6b) of diabetic mice. These results combined with *in vitro* results suggested that WS-PE exerted antidiabetic effects partly by stimulating the expression and translocation of GLUT4.

### WS-PE stimulated expression of GLUT4 and phosphorylation of GSK3*β* by activating Akt in vivo

**Fig. 7:**
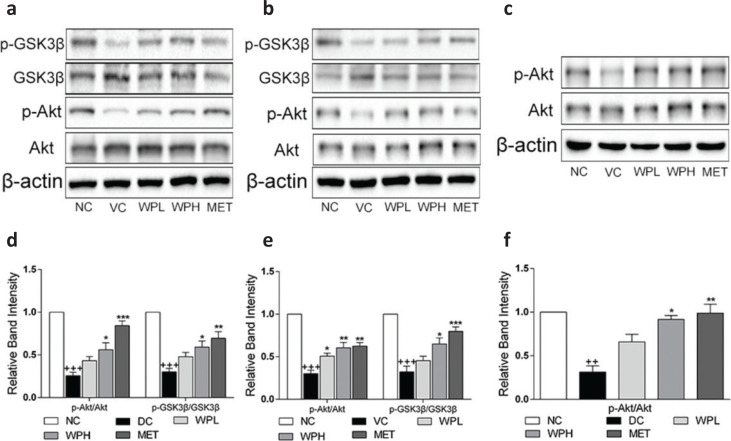
The WS-PE treatment stimulated Akt phosphorylation in WAT, liver, and skeletal muscles, and WS-PE increased p-GSK3β expression in skeletal muscles and liver. Western blot analysis in skeletal muscles (a), liver (b), and WAT (c). Relative band intensity of western blot for skeletal muscles (d), liver (e), and WAT (f). Data are means ± SEM, shown as relative band intensity compared with NC group (*n* = 3). ^+++^ *P* < 0.01 compared with NC group, ^**^ *P* < 0.01 compared with DC group. NC: normal control group. DC: diabetic control group. WPL: a low-dose of WS-PE-treated group. WPH: a high-dose of WS-PE-treated group. MET: metformin-treated group.

Phosphorylation of Akt was detected in insulin-sensitive tissues *in vivo*. Results showed that the ratio of p-Akt/Akt was lower in diabetic mice in the DC group compared with the NC group mice. Interestingly, 4-week treatment with WS-PE or metformin increased p-Akt expression and enhanced the ratio of p-Akt/Akt in skeletal muscles (Fig. 7a, d), the liver (Fig. 7b, e), and WAT (Fig. 7c, f). GSK3β, a key downstream target of Akt, is greatly influential in regulating glycogen synthesis through phosphorylation. The ratio of p-GSK3β/GSK3β in the liver and skeletal muscles of diabetic mice was decreased in the DC group compared to the NC group (Fig. 7a, b). However, diabetic mice treated with WS-PE or metformin showed increased ratios of p-GSK3β/GSK3β, suggesting that WS-PE potentially had a role in regulating glycogen synthesis (Fig. 7a, b, d, e). These results were consistent with our *in vitro* results, suggesting a hypothesis that WS-PE regulates disorderly glucose and lipid metabolism by activating Akt.

## Discussion

In this study, WS-PE, an extract isolated from edible flowers of *W. sinensis*, was investigated to explore antidiabetic effects and potential mechanisms both *in vivo* and *in vitro*. The results obtained from HFD + low-dose STZ-induced diabetic mice and L6 cells showed that WS-PE exerted antidiabetic effects *via* the activation of Akt/GLUT4 and Akt/GSK3β and further improvement of hyperglycemia, IR, and dyslipidemia.

GLUT4, a key regulator for transporting glucose into cells, is important for glucose utilization and IR (22). When carbohydrates are ingested, insulin stimulates GLUT4 translocation from an intracellular vesicle to the cytomembrane, and GLUT4 further facilitates the intracellular transport of glucose (22). Published reports have shown that translocation and expression defects of GLUT4 are hallmarks of peripheral IR (23).

A cell-based GLUT4 translocation assay system with L6 cells that stably over-expressed IRAP-mOrange was established to look for potential functional foods that stimulate GLUT4 translocation. WS-PE promoted GLUT4 translocation *in vitro* (Fig. 1a, b). Furthermore, WS-PE significantly stimulated GLUT4 expression and increased glucose uptake in L6 cells (Fig. 1c–e). The main signaling pathways including AMPK/GLUT4, Akt/GLUT4, and PKC/GLUT4 are reported responsible for regulating GLUT4 expression and translocation (24–26). As shown in Fig. 1d and e, GLUT4 expression was enhanced with increasing levels of Akt phosphorylation, suggesting that WS-PE stimulates GLUT4 expression by activating the Akt/GLUT4 pathway. These results are in agreement with previous reports and preliminarily conclude that WS-PE may have antidiabetic effects that are exerted by targeting Akt/GLUT4 (27). GSK3β, a well-known crucial downstream target of Akt, exerts significant effects on regulating glycogen synthesis. The phosphorylation of GSK3β inhibits the phosphorylation of GS (glycogen synthase) to stimulate glycogen synthesis (28). WS-PE enhanced the ratio of p-GSK3β/GSK3β in L6 cells, which was consistent with the increasing level of Akt phosphorylation (Fig. 1e). These results indicated that WS-PE increased glucose uptake *in vitro* by activating Akt and further influencing GLUT4 and phosphorylation of GSK3β.

To investigate antidiabetic effects and the potential underlying mechanism of WS-PE *in vivo*, HFD + low-dose STZ-injection induced type 2 diabetic mice were used as the animal model in the present study. These type 2 diabetic mice are known to share major characteristics with human T2DM, including hyperglycemia, body weight loss, IR, and lipid metabolism disorders (29, 30). It is well-known that long-term HFD leads to obesity, IR, and hyperinsulinaemia, and then, the subsequent injection of low-dose STZ, a beta cell toxin, would partly damage pancreatic β-cells and induce body weight loss and high levels of FBG (31). Mice in the DC group showed hyperglycemia, lipid metabolism disorders, and high serum insulin levels, indicating that the diabetic mice model was successfully created. The regulation of insulin secretion and blood glucose level at a safe level are two primary goals of diabetic treatment, which avoid extreme diabetic complications (32). In the *in vivo* study, high FBG levels of diabetic mice were ameliorated significantly with the WS-PE treatment (Fig. 2a), and the damaged glucose tolerance was reversed (Fig. 2c). Moreover, serum insulin levels of diabetic mice, which are an intuitive indicator of IR, decreased with the WS-PE treatment (Fig. 3a). These results showed that WS-PE had a great role in ameliorating hyperglycemia and improving IR *in vivo*. Diabetes is characterized by body weight loss because high blood glucose levels inhibit nutrient absorption and too much urination expels the nutrients (33). Diabetic mice showed obvious body weight loss, but normal mice fed with normal diet maintained normal body weight. After 4-week treatment with WS-PE or metformin, it was seen that body weight loss was reasonably well controlled in mice in the WS-PE-treated group (Fig. 2b). In general, WS-PE ameliorated high levels of FBG, damaged OGTT, body weight loss, and IR.

According to previous reports, lipid dyslipidemia is characterized by high levels of LDL-C, TG, TC, and FFA and low levels of HDL-C; these are widely acknowledged as factors inducing IR and T2DM (34). WS-PE resolved dyslipidemia in diabetic mice, and the levels of several serum lipid parameters, including LDL-C, TG, TC, FFA, and HDL-C were improved to different degrees (Fig. 3). Moreover, hepatic steatosis of diabetic mice was reversed by the WS-PE treatment (Fig. 4), indicating that WS-PE ameliorated the lipid accumulation and abnormal lipid metabolism disorders in mice. ALT and AST are known as markers of hepatic lipotoxicity and are used to evaluate the degree of hepatic steatosis damage (35). The WS-PE treatment decreased the serum levels of ALT and AST (Fig. 3g, h), which further proved that hepatic steatosis was reversed in diabetic mice. Taken together, WS-PE ameliorated lipid metabolism disorders in many ways to further improve IR and T2DM.

GLUT4 is mainly present in adipose tissues, skeletal muscles, and cardiac muscle cells, and GLUT4 translocation and expression levels are related to insulin-regulated glucose homeostasis *in vivo*, which also have profound effects on metabolic disturbances of glucose and lipids in the state of IR (36, 37). Some published reports have showed that glucose transportation disorders of WAT and skeletal muscles were associated with reduced GLUT4 expression (38). In the present study, skeletal muscles and WAT were selected to evaluate the positive effects of WS-PE on GLUT4 expression; both these tissues are important insulin-sensitive tissues and are directly related to glucose transport and utilization *in vivo* (39, 40). Results showed that 4-week treatment of WS-PE increased the GLUT4 expression in WAT and skeletal muscles of diabetic mice (Fig. 6).

Akt, also known as protein kinase B, has a key role in glucose and lipid metabolism and has attracted increasing interest (41). Previous reports have shown that Akt activation in skeletal muscles, liver, and WAT enhanced glucose utilization and regulated lipid metabolism disorders in various ways (41). Western blot analysis for insulin-sensitive tissues showed that WS-PE increased Akt phosphorylation in skeletal muscles, liver, and WAT, indicating that WS-PE exerted antidiabetic and antidyslipidemic effects by activating Akt *in vivo* (Fig. 7). We also concluded that WS-PE increased GLUT4 expression by Akt activation because Akt activation stimulated GLUT4 expression. GSK3β plays a key role in regulating glycogen synthesis and gluconeogenesis in the liver and skeletal muscles (28). p-GSK3β was increased in the liver and skeletal muscles, indicating that WS-PE regulates hepatic glycogen synthesis and muscle glycogen synthesis to affect glucose homeostasis (Fig. 7). These results were consistent with the above *in vitro* results and further verified that WS-PE has potential antidiabetic effects by targeting Akt/GLUT4 and Akt/GSK3β *in vivo* and *in vitro*.

In conclusion, WS-PE, an extract isolated from edible flowers of *W. sinensis*, has potential antidiabetic effects. *In vitro*, WS-PE stimulated glucose uptake in L6 cells by activating Akt, further increasing GLUT4 translocation and expression, and enhancing p-GSK3β expression. *In vivo,* the WS-PE treatment ameliorated several anomalies in T2DM mice, including hyperglycemia, body weight loss, glucose intolerance, hyperinsulinemia, dyslipidemia, hepatic steatosis, and pancreatic islet destruction. Furthermore, WS-PE increased GLUT4 expression in skeletal muscles and WAT, stimulated p-Akt expression in skeletal muscles, the liver, and WAT, and enhanced GSK3β phosphorylation in the liver and skeletal muscles. Overall, WS-PE acts as an antidiabetic agent by targeting Akt/GLUT4 and Akt/ GSK3β.

## Ethics and consent

After getting authorization from the Animal Ethics Committee of SCUN (Approval Number: S08917111E), the use and care of animals and all procedures involving animals were carried out according to the Guidelines for Animal Experiments of SCUN.

## Supplementary Material

Antidiabetic effects of a lipophilic extract obtained from flowers of *Wisteria sinensis* by activating Akt/GLUT4 and Akt/GSK3β
