# Supplementary material for: Antidiabetic effects of a lipophilic extract obtained from flowers of Wisteria sinensis by activating Akt/GLUT4 and Akt/GSK3β
Source: Food Nutr Res. 2020 Sep 7;64:10.29219/fnr.v64.3589. doi: 10.29219/fnr.v64.3589 (PMC7534953; doi:10.29219/fnr.v64.3589)
Supplement: Antidiabetic effects of a lipophilic extract obtained from flowers of Wisteria sinensis by activating Akt/GLUT4 and Akt/GSK3β [file FNR-64-3589-s001.docx]

**Supplementary Material**

**S1. Screening methodology validation**

There are several papers reported that IRAP-mOrange and GLUT4-eGFP could be applied to detect the GLUT4 translocation in L6 (1-3) and 3T3-L1 cells (4, 5). In order to validate the feasibility of our IRAP translocation assay for discovering potential hypoglycemic agents, we have observed the effects when the GLUT4-eGFP or IRAP-marked L6 cells treated with insulin and berberine which are definitely pharmacodynamic GLUT4 agonists. L6 cells which stably express IRAP-mOrange and GLUT4-eGFP were cultured in α-MEM supplemented with 10% fetal bovine serum and 1% antibiotics (100 U/mL penicillin and 100 μg/mL streptomycin) at 37 ^o^C in 5% CO2. L6 cells was seeded in 48 well plates, and incubated until 100% confluence and then starved in serum-free MEM-ɑ for 2 h. Afterwards, L6 cells were treated with insulin (10 nM) and berberine (5 μM). The cells were taken photos with a laser-scanning confocal microscope LSM 510 (Carl Zeiss, Jena, Germany) to supervise the IRAP-mOrange and GLUT4-eGFP translocation. And the images were captured with 555 nm excitation laser every 10 seconds in first 5 minutes and then every 5 minutes in later 30 minutes.

During the experiment, as time went on, we could observe the green and red fluorescence enhanced significantly after treating with insulin and berberine in L6 cells (Fig S1). The results showed that GLUT4 and IRAP simultaneously translocated onto the plasma membrane in 30 min when adding the GLUT4 agonist. GLUT4 has mainly been recruited to the PM throughout to the GLUTs storage vesicles (GSV). Three main proteins stored in GSV are GLUT4, IRAP, and Sortilin (6). It was reported that IRAP and GLUT4 displayed a strong colocalization (7, 8) in many researches. Thus, detecting the IRAP can indirectly reflect the situation of GLUT4. So our results could be explained that detecting the IRAP-mOrange fluorescence could indirectly reflect the GLUT4 translocation. As the red fluorescence is more conspicuous than green fluorescence for observation, so we choose the IRAP-mOrange fluorescence assay for reflecting GLUT4 translocation.

**A B**


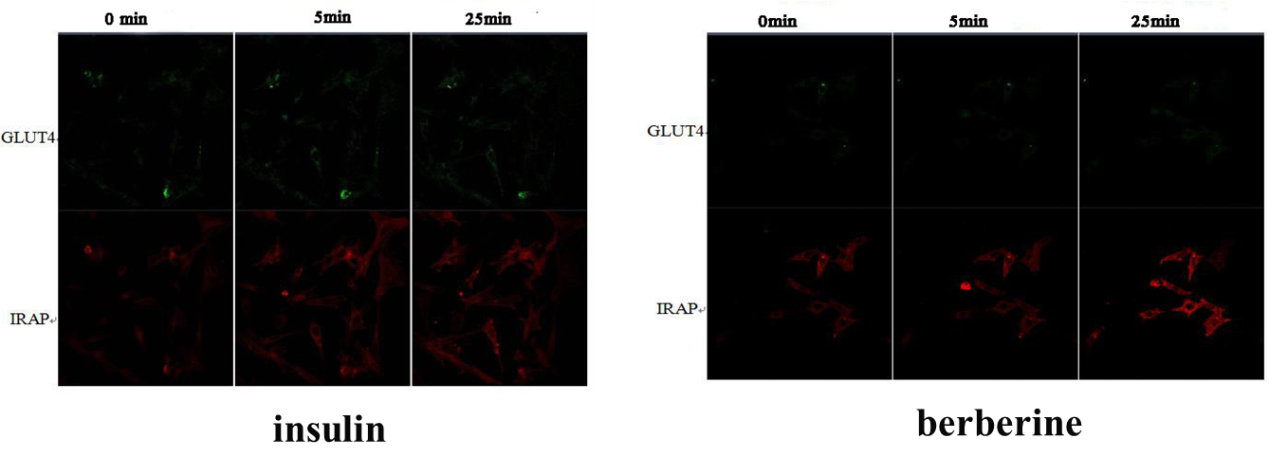


**Figure S1** L6 cells were infected with IRAP-mOrange and GLUT4-eGFP in order to detect externalized GLUT4 translocation by confocal microscopy. (A) Confocal images in L6 cells incubated in the absence (0 min) or presence of insulin for 5min, 30 minutes. (B) Confocal images in L6 cells incubated in the absence (0 min) or presence of berberine for 5 min, 30 minutes.

**References**

1. Wang X, Qu F, Chen Z, Liang T, Qu A. Labeling and imaging of GLUT4 in live L6 cells with quantum dots. Biochem Cell Biol. 2009;87(4):687-94. doi:10.1139/o09-041

2. Zhou Q, Yang X, Xiong M, Xu X, Zhen L, Chen W, et al. Chloroquine Increases Glucose Uptake via Enhancing GLUT4 Translocation and Fusion with the Plasma Membrane in L6 Cells. Cell Physiol Biochem. 2016;38(5):2030-40. doi:10.1159/000445562

3. Huang M, Zhao P, Xiong M, Zhou Q, Zheng S, Ma X, et al. Antidiabetic activity of perylenequinonoid-rich extract from Shiraia bambusicola in KK-Ay mice with spontaneous type 2 diabetes mellitus. J Ethnopharmacol. 2016;191:71-81. doi:10.1016/j.jep.2016.06.018

4. Bai L, Wang Y, Fan J, Chen Y, Ji W, Qu A, et al. Dissecting multiple steps of GLUT4 trafficking and identifying the sites of insulin action. Cell Metab. 2007;5(1):47-57. doi:10.1016/j.cmet.2006.11.013

5. Jiang L, Fan J, Bai L, Wang Y, Chen Y, Yang L, et al. Direct quantification of fusion rate reveals a distal role for AS160 in insulin-stimulated fusion of GLUT4 storage vesicles. J Biol Chem. 2008;283(13):8508-16. doi:10.1074/jbc.M708688200

6. Shi J, Kandror KV. Sortilin is essential and sufficient for the formation of Glut4 storage vesicles in 3T3-L1 adipocytes. Dev Cell. 2005;9(1):99-108. doi:10.1016/j.devcel.2005.04.004

7. Kumar A, Lawrence JC, Jr., Jung DY, Ko HJ, Keller SR, Kim JK, et al. Fat cell-specific ablation of rictor in mice impairs insulin-regulated fat cell and whole-body glucose and lipid metabolism. Diabetes. 2010;59(6):1397-406. doi:10.2337/db09-1061

8. Rubin BR, Bogan JS. Intracellular retention and insulin-stimulated mobilization of GLUT4 glucose transporters. Vitam Horm. 2009;80:155-92. doi:10.1016/S0083-6729(08)00607-9
